# Loss of tumor suppressor inositol polyphosphate 4-phosphatase type B impairs DNA double-strand break repair by destabilization of DNA tethering protein Rad50

**DOI:** 10.1038/s41419-020-2491-3

**Published:** 2020-04-27

**Authors:** Yue Sun, Xuelian Ning, Jiankun Fan, Jiandong Hu, Yanting Jiang, Ziqi Hu, Joao A. Paulo, Jichao Liu, Xiaohong Qiu, Hui Xu, Songbin Fu, Steven P. Gygi, Jinwei Zhang, Chunshui Zhou

**Affiliations:** 1 The Laboratory of Medical Genetics, Harbin Medical University, Harbin, 150081 China; 2 Department of Cell Biology, Harvard Medical School, Boston, MA 02115 USA; 3 The 2th Affiliated Hospital, Harbin Medical University, Harbin, 150001 China; 4 The Tumor Hospital, Harbin Medical University, Harbin, 150081 China; 5 Key Laboratory of Preservation of Human Genetic Resources and Disease Control in China (Harbin Medical University), Ministry of Education, Harbin, 150081 China

**Keywords:** Oncogenes, Cell signalling

## Abstract

Genome instability is the fundamental hallmark of malignant tumors. Tumor suppressors often play a role in maintaining genome stability. Our previous genetic screen identified inositol polyphosphate 4-phosphatase type B (INPP4B), primarily hydrolyzing phosphatidylinositol 3, 4-disphosphate, is a potential tumor suppressor in lung cancer cells. How INPP4B regulates the genome stability of lung cancer cells is unclear. Here we report knockout of INPP4B in lung adenocarcinoma A549 cells by Crispr-Cas9 gene editing leads to sensitization to ionizing radiation (IR), PARP inhibitor olaparib and impaired DNA homologous recombination repair. Re-introduction of a Crispr-Cas9 resistant INPP4B gene in the INPP4B knockout cells partially restored their resistance to IR, indicating loss of INPP4B protein is relevant to the increased IR sensitivity. Furthermore, we showed ectopic expressed INPP4B in A549 cells responds to IR irradiation by redistribution from cytoplasm to nucleus and endogenous INPP4B protein interacts with Rad50, a crucial MRN complex component for tethering DNA double-strand breaks. Loss of INPP4B protein results in decreased stability of Rad50 in vivo, suggesting an unanticipated role of tumor suppressor INPP4B in maintaining genome integrity via facilitating Rad50 mediated DNA double-strand break repair. Taken together, our findings support a dual role of INPP4B in suppression of tumorigenesis by safeguarding genome stability, as well as inhibiting of PI3K-Akt-mTOR signaling, and offer a new therapeutic strategy for personalized cancer treatment to patients with INPP4B defects or deficiency in the clinic.

## Introduction

Genome instability is the fundamental hallmark of human malignancies^1^, many gene mutations and large genomic alterations including deletions, translocations, loss of heterozygosity and amplifications are casual factors for tumorigenesis. DNA damage generated by exogenous genotoxic insults, endogenous replication errors and intrinsic defects of DNA repair are the main culprit for genome instability. Therefore, targeting genome integrity maintenance is a major anti-cancer mechanism^2^. Tumor suppressors, more often, play an important role in maintaining genome stability. Based on their functions in genome maintenance, two broad categories of tumor suppressors could be classified. The first category including TP53, p21, Rb, and p16, etc. serves as a genome gatekeeper and guardian, they usually induce cell cycle arrest, transcriptional reprogramming and apoptosis or cellular senescence under genotoxicity or genomic insults to protect genome integrity. The second category including BRCA1, BRCA2, MLH1, MSH2, etc. acts as a caretaker and directly participates in DNA damage repair process to prevent genome from alterations^3^.

Accumulating evidence indicates that enzymes involved in the metabolism of phosphate inositol and a number of inositol polyphosphates directly involve in DNA damage repair and genome stability maintenance. For example, loss of PTEN, a well-known tumor suppressor and a lipid phosphatase antagonizing PIK3-Akt mTOR activation by dephosphorylating phosphoinositide-3, 4, 5-trisphosphate, plays a critical role in the maintenance of chromosomal stability through the physical interaction with centromeres and control of Rad51 mediated double-strand DNA repair^4,5^. Inositol hexakisphosphate kinase 1 (IP6K1) is required for homologous recombination (HR) repair in mammalian cells^6^. Kcs1, an ortholog of IP6K1 in *S. cerevisiae*, is essential for budding yeast resistance to DNA damaging agent methyl methanesulfonate^7^. Moreover, inositol hexakisphosphate (IP6), the substrate of IP6K1, was found to bind Ku70/80 and stimulate non-homologous end-joining (NHEJ) repair process^8,9^. In addition, IP6 was also demonstrated to mediate the interaction of cullin-COP9 signalosome with Cullin-RING E3 Ligases (CRLs) to inhibit the activity of CRLs^10^. On the contrary, IP6K1 was found to convert IP6 into IP7 and release CRL4 and initiate the UV-elicited nucleotide excision repair (NER)^11^.

In a previous genetic screen for transformation suppressors in human immortalized lung epithelial cell BEAS-2B, we identified inositol polyphosphate 4-phosphatase type B (INPP4B) as a candidate lung tumor suppressor by suppression of PIK3-AKT-mTOR signaling via dephosphorylating phosphatidylinositol 3, 4-disphosphate^12^. Loss or low expression of INPP4 is often found in lung, breast, ovarian, prostate, as well as many other cancers^13–15^. Furthermore, INPP4B has been implicated in regulation of DNA damage sensitivity in a number of cancer cell lines including breast cancer cell line MDA-MB-231, leukemia cell line KG-1 and ovarian cancer cell line Ovca429^16–18^. In order to elucidate the molecular mechanism of how INPP4B safeguards genome integrity in lung cancer A549 cells, we knocked out INPP4B in these cells by employing Crispr-Cas9 gene editing and discovered an unexpected role of INPP4B in DNA repair by maintaining the stability of Rab50, a critical component of Mre11/Rad50/Nbs1 complex for sensing and tethering DNA double strand breaks in mammalian cells.

## Materials and methods

### Cell culture and DNA damage treatment

Human cancer cell lines A549 was purchased from ATCC and cultured in F12K medium supplemented with 10% fetal bovine serum, 100 IU/mL of penicillin and streptomycin and in an incubator with 5% CO_2_ at 37 °C. For DNA damage treatment, cells were subjected to 0.5uM olaparib or ionizing radiation (gamma ray, Co-60 source).

### Antibody and chemical reagents

All chemicals including insulin, G418, puromysin, hygromycin B were from Sigma-Aldrich. Porcine sequencing grade trypsin was from Promega (Fitchburg, USA), olaparib was from MCE (Monmouth Junction, USA). Antibodies against INPP4B(#8450),Akt(#9272), phospho-Akt-S473(#4060), glyceraldehyde-3-phosphate dehydrogenase (GAPDH)(#97166), phospho-p70 S6 kinase(#9206), phospho-histone H2AX (Ser139)(#3014) were from Cell Signaling Technology, Rad50(MA1-23269) antibody was from Invitrogen (Carlsbad, USA) and Rad51(PC130) antibody from Calbiochem (Billerica, USA), Flag M2(F1804) antibody was from Sigma-Aldrich (St Louis, USA). Secondary antibodies conjugated with FITC were from Rockland (Limerick, USA). For Western blotting, antibodies were used at a dilution of 1:1000, for immunofluorescence staining, antibodies were used at 1:400.

### Plasmid construction and cell line generation

The Crispr-INPP4B cell line was generated by transfection of a Lenti-Crispr V2 vector expressing a gRNA targeting INPP4B into A549 following 1 μg/mL puromysin selection. The shINPP4B A549 cells were generated with transfection of an MSCV-Hyg plasmid expressing small hairpin RNA targeting INPP4B mRNA following 200 μg/mL hygromycin B selection. The rescued A549 cell line was created by transfection of pcDNA 3.1 3XFlag vector expressing a Crispr resistant INPP4B ORF into the knockout cells. The control cell line was established by co-transfection of empty Lenti-Crispr V2 vector or/and MSCV-Hyg plasmid.

A gRNA (5′-tgaaaacacagccaaagcaa-3′) targeting INPP4B 13^TH^ exon was cloned into Lenti-Crispr V2 vector (a gift from Zhang Feng lab, MIT) via BsmBI restriction sites. For gene knockdown, primer targeting INPP4B (5′-gctcctgtccgtgatcgtaa-3′) and primer targeting Akt (5′-gagtttgagtacctgaagct-3′) were inserted into MSCV-Hyg plasmid (a gift from Stephen Elledge, Harvard) through XhoI and EcoRI sites, the primer targeting firefly luciferase gene (5′-cgcctgaagtctctgatta-3′) (shFF2) was used as negative shRNA control. For INPP4B rescue experiment, a Crispr resistant INPP4B ORF was generated by site-directed mutagenesis with following two primers containing nucleotide mutations in the gRNA targeting region without affecting their coding amino acids: Crispr-INPP4B-resis-F: 5′-cctgaaaagaccggtaagggtaaggaagttc-3′; Crispr-INPP4B-resis-R: 5′–gaacttccttacccttaccggtcttttcagg-3′ and cloned into pcDNA 3.1 3xFlag vector through XhoI and KpnI sites. Wide type INPP4B ORF was cloned into pEGFP-C1 through XhoI and KpnI for over-expression of GFP-INPP4B.

### Cell proliferation and survival assay

Cells were plated at a density of 5000 cells per well in 96-well plates. After 24 h of culture, the medium was removed and MTT assay was performed according to the manual (Invitrogen). Absorbance at 495 nm was recorded by a plate reader (Tecan sunrise, Switzerland). Cell growth was determined following normalization and comparison with the cell absorbance at day 1. For cell survival assay post IR irradiation, survival rates at the desired time points were determined by cell counting and normalized to the untreated cells immediately prior to IR.

### Fluorescence activated cell sorting (FACS)

To detect apoptosis, cells with appropriate treatments were harvested by trypsin digestion and incubated with Annexin V-FITC conjugates in PBS buffer containing 1 mg/ml propidium iodide and 0.1 mg/ml RNase A. After incubation in dark, cells were subjected to FACS (BD Biosciences). To detect GFP expression, the desired cells were trypsinized and resuspended in PBS solution and subjected to FACS.

### Immunostaining for DNA damage foci

The desired cells were cultured on coverslips. After DNA damage treatment, cells were fixed in 4% (wt/vol) paraformaldehyde for 20 min at room temperature, permeabilized in 0.2% Triton X-100 for 30 min, blocked with 5% (wt/vol) bovine serum albumin and incubated with the primary antibodies (Rad51 or γ-H2AX) overnight and followed by incubating with secondary antibodies conjugated with FITC labeling (Rockland) for 1 h at room temperature. Coverslips were mounted in 4′,6-diamidino-2-phenylindole (DAPI) containing media (Vectashield, Burlingame, USA). Signals were visualized using a Leica DM500 B fluorescence confocal microscope (Wetzlar, Germany).

### Immunoprecipitation for Western blotting or mass spectrometry

Large scale immuno-precipitations of INPP4B in A549 cells were performed as described previously^19^. A549 cells were harvested by scraping and lysed with RIPA buffer containing 50 mM Tris (pH 7.4), 150 mM NaCl, 1 mM EDTA, 0.25% sodium deoxycholate, proteinase inhibitor cocktail (Roche, Switzerland) for 20 min at 4 °C. The lysate mixture was subjected to sonication to completely disrupt cells. Lysates were clarified by centrifugation at 14,000 rpm for 30 min at 4 °C. One microgram of antibodies (20 μg Ab for large scale IP) against endogenous INPP4B or Rad50 or an unrelated antibody and 20 μl of magnetic protein A beads slurry (100 μl for large scale IP) were used for a single IP in a volume of 1 ml containing 2 mg proteins (or 40 ml containing 100 mg proteins for large scale IP). IP reactions were performed at 4 °C for 4 h with gentle rotation. Bound proteins on beads were washed with RIPA buffer for three times. The proteins remained on the beads were eluted by boiling in 100 μl of 1× SDS-PAGE sample loading buffer, and resolved on SDS–PAGE gel. The resulting gels were either subject to Western blotting or coomassie staining, in-gel trypsin digestion and mass spectrometry identification.

### Protein half-life measurement

Cells at log phase were seeded into 6 cm plates at 3 × 10^5^ cells per plate. After one day of culture, 50 μg/ml of CHX was added into each plate, and cells were harvested at a 10 min interval for a 1 h period of time course. The whole-cell lysates were prepared by adding 200 μl of 2× SDS loading buffer to each plate, and the lysates were collected and subjected to sonication. Twenty microliter of each cell lysate were loaded and resolved on 6% SDS–PAGE gels, antibodies against Rad50 were used for probing endogenous Rad50. The Western blot signals were quantified by densitometry scanning and normalized to GAPDH signal. The mean Rad51 half-life was based on three independent CHX treatments and expressed as means ± SD (min).

### Statistical analysis

ALL analyses were performed using SPSS for Windows 17.0 software (SPSS Inc, Chicago, USA). A one-way ANOVA test or a Student’s t-test was used for comparison of cell proliferation, cell survival, apoptosis and foci formation, *p* value less than 0.05 is considered statistically significant.

## Results

### Loss of INPP4B causes sensitization to ionizing radiation, such sensitization is enhanced by DNA repair inhibitor olaparib

**Fig. 1:**
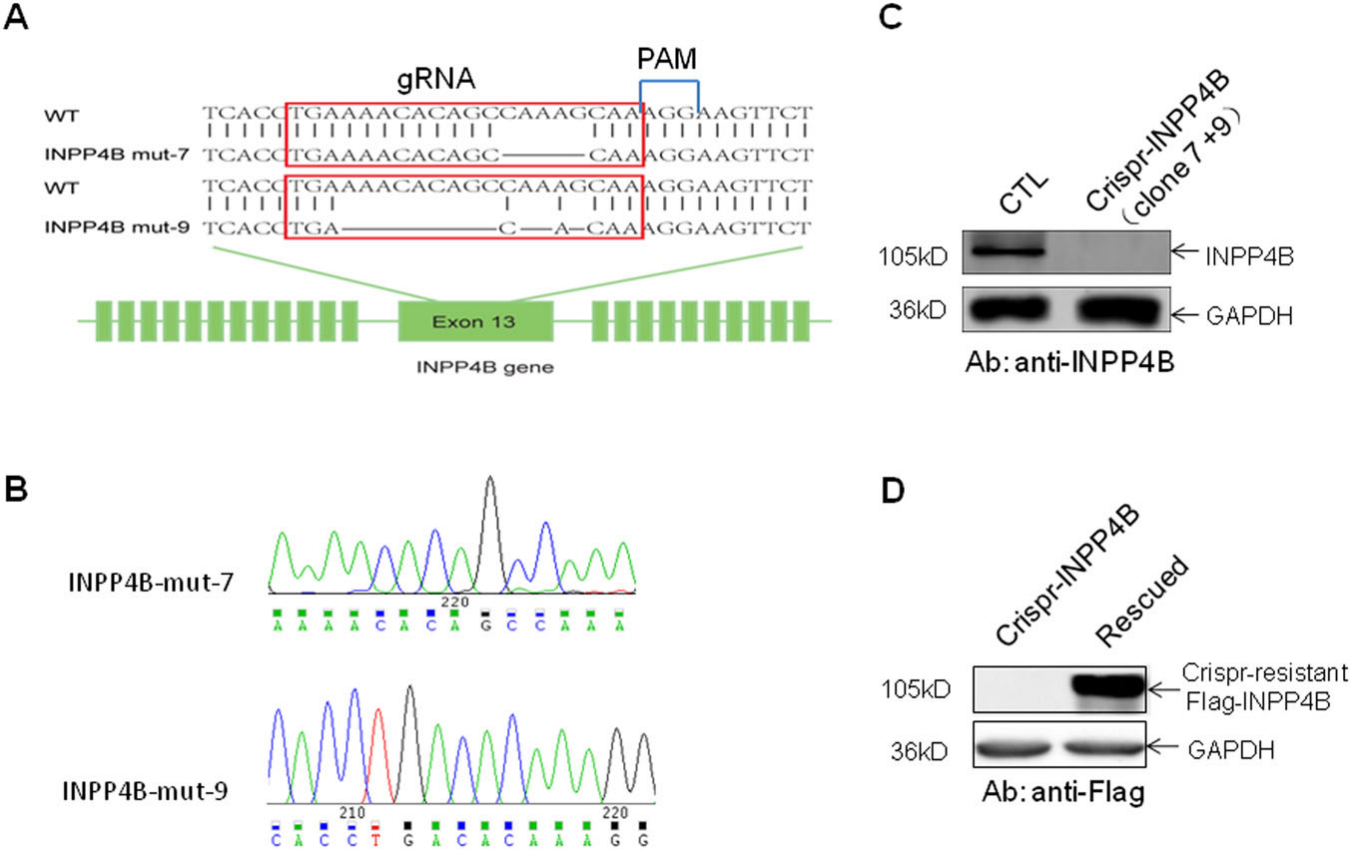
Generation and confirmation of INPP4B knockout A549 cell lines by Crispr-Cas9 gene editing. **a** The gRNA sequence targeting the 13th exon of wild type (WT) INPP4B allele is highlighted with red frames and aligned with two INPP4B mutant alleles carrying reading frame shift mutations created by CRISPR-Cas9 editing in lung adenocarcinoma A549 cells, blank regions indicate the deleted nucleotides. **b** Sanger-sequencing chromatograms of the gRNA targeting regions from the two INPP4B mutant alleles created by Crispr-Cas9 technology, the nucleotide sequence is shown under each chromatogram. **c** Complete loss of INPP4B protein expression in Crispr-INPP4B A549 cells was confirmed. Note: the INPP4B knockout cells are a mixture of clone 7 and clone 9. **d** The expression of a 3xFlag tagged Crispr-resistant INPP4B was verified in Crispr-INPP4B A549 cells.

**Fig. 2:**
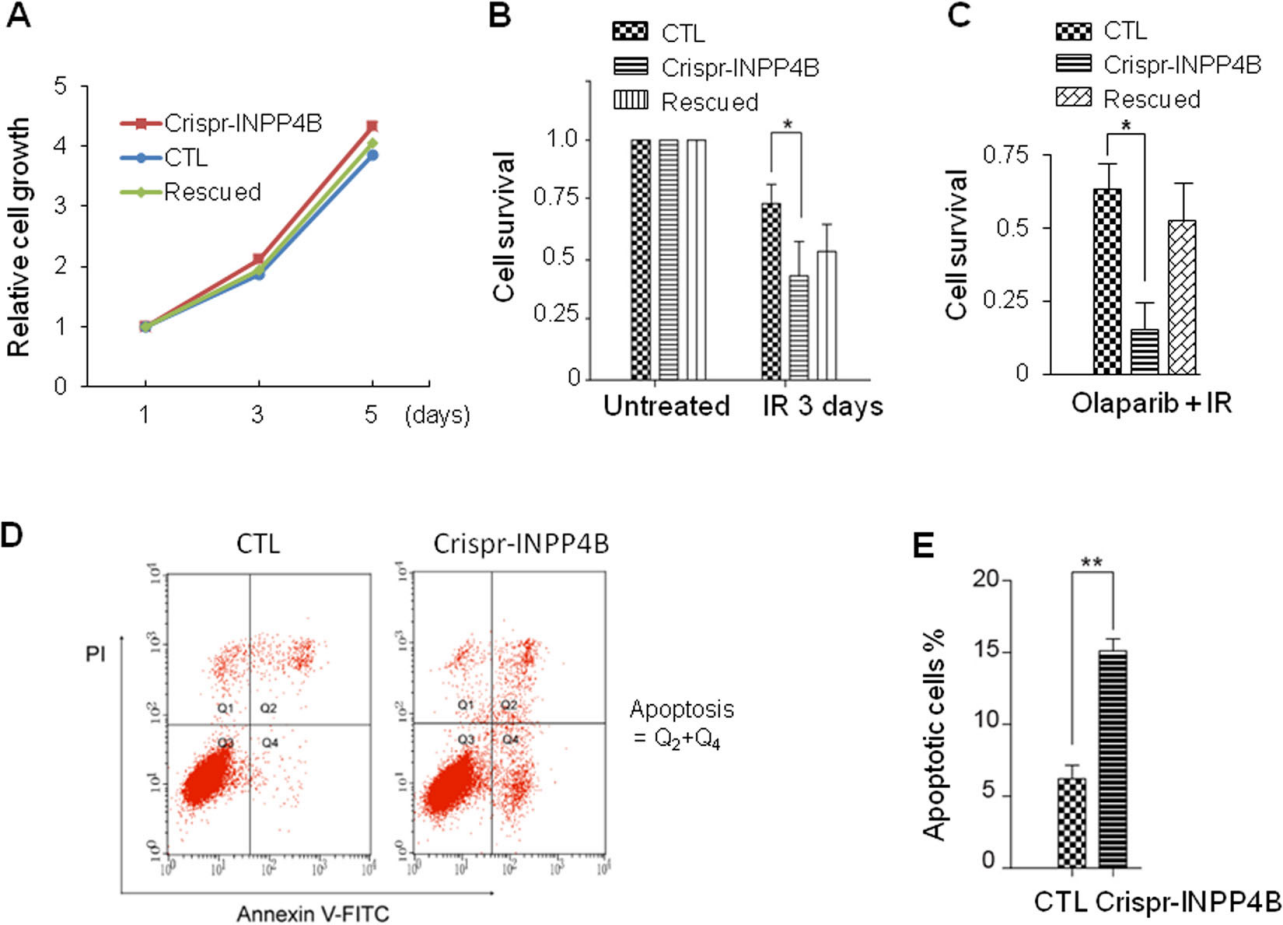
Knockout of INPP4B in A549 cells leads to sensitization to IR irradiation and PARP inhibitor olaparib. **a** Under normal culture condition, knockout of INPP4B does not affect cell proliferation. Cell growth rates were measured and calculated from three independent MTT assays. **b** The cell survival rate was decreased in Crispr-INPP4B cells following IR. The indicated cell lines were seeded into 3.5 cm plates at 3 × 10^5^/plate. After one day of culture, cells were irradiated with 20 Gy of IR, survival cells were counted on day 3 post IR, the average survival rates were normalized to the untreated cells immediately prior to IR irradiation and plotted in **b**. Data were presented as average survival rates ± SD from 3 independent experiments. *p < 0.05 indicates a significant difference of cell survival between CTL and Crispr-INPP4B cells by one-way ANOVA test. **c** DNA repair inhibitor PARP enhanced the IR sensitivity of INPP4B knockout cells. The three cell lines used in **b** were pretreated with 0.5 μM olaprib for 4 h and subjected to 20 Gy of IR. Survival cells were counted on day 3. **d** Cells on day 3 post IR were trypsin digested and harvested for Annexin V-FITC staining. Cells were analyzed by fluorescence activated cell sorting shown in **d**, and the average percentage of each group of apoptotic cells from 3 independent IR treatments was plotted in **e**. **(*p* < 0.01 by Student’s *t* test) indicates a significant survival difference between CTL and Crispr-INPP4B cells.

In our pilot functional screen for transformation suppressors in lung epithelial cell BEAS-2B, inositol polyphosphate 4-phosphatase type B (INPP4B) gene was identified^12^. In order to elucidate the molecular mechanism of INPP4B in regulating genome integrity in lung cells, we generated INPP4B knockout cell lines by Crispr-Cas9 gene editing technology targeting the exon of INPP4B gene in lung adenocarcinoma cell line A549 (Fig. 1A). Two cell clones were generated and verified by DNA sequencing with deletions occurred in the gRNA targeting region, resulting in two INPP4B mutant genes with reading frame shift mutations (Fig. 1B). The expression of intact INPP4B protein was completely lost in both clones confirmed by Western blotting against endogenous INPP4B protein (Fig. 1C). We mixed the two cell clones and designated them as Crispr-INPP4B A549 cell line hereafter. In parallel, we used the A549 cells transfected with a Lenti-crispr V2 empty vector as the control cell line and named as CTL A549. In addition, to exclude the off-target effect of Crispr-Cas9, we also introduced a Crispr-resistant INPP4B gene driven by pcDNA 3.1-3xFlag vector into the Crispr-INPP4B A549 cell line and named as rescued A549 (Fig. 1D). First of all, we compared the proliferation rate of the three cell lines by MTT assay. We found knock out of INPP4B does not affect A549 cell growth under normal culture condition (Fig. 2A). Surprisingly, when these cells were exposed to 20 Gy IR irradiation (gamma ray), we found the cell survival rates for CTL and Crispr-INPP4B cells were 73.6 ± 7.9% and 43.5 ± 14.2% on day 3 post IR, respectively, the growth difference (*p* < 0.05) indicates IR sensitization upon loss of INPP4B. Meanwhile, re-introduction of a Crispr-resistant-INPP4B gene in the rescued cells partially restored the resistance to IR irradiation, indicating the observed sensitization to IR is related to the loss of INPP4B expression (Fig. 2B). Furthermore, pretreatment of the three cell lines with PARP inhibitor olaparib could significantly enhance the IR sensitization in Crispr-INPP4B cells, but not in CTL and rescued cells (Fig. 2C). Given the fact that olaparib only generates synthetic lethality through the combination of two molecular pathways^20,21^, thus, sensitization to olaparib hints an intrinsic DNA repair defect occurred in the INPP4B knockout A549 cells.

Next, we determined the type of cell death occurred in these cells following 20 Gy IR. CTL, Crispr-INPP4B and the rescued cells were harvested on day 3 after IR, stained with Annexin V-FITC conjugates and subjected to the fluorescence activated cell sorting (FACS). We found the apoptotic population in Crispr-INPP4B cells (15.13 ± 0.83%) is much higher than that in CTL cells (6.23 ± 0.93%, *p* < 0.01), demonstrating apoptosis is the major type of cell death in the IR treated A549 cells with loss of INPP4B (Figs. 2D and 2E).

### Elevated activation of Akt does not contribute to the DNA damage sensitization induced by loss of INPP4B

**Fig. 3:**
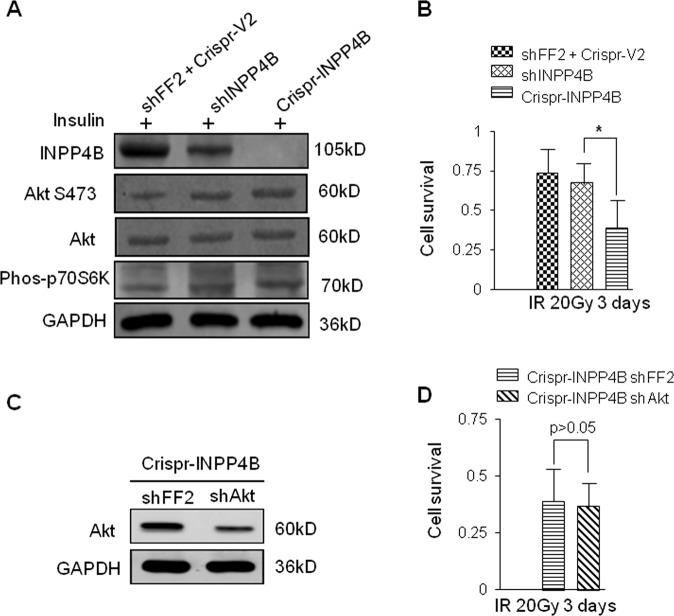
Activation of Akt is not major cause for IR sensitization in INPP4B knockdown cells. **a** A similar level of AKT activation occurred in both INPP4B knockdown and INPP4B knockout cells. Each cell line was serum starved for 72 h, followed by addition of 100 nM insulin for 20 min. Cells were harvested, phosphorylation of Akt, p70S6K and expression of INPP4B were determined by Western blotting, GADPH was served as loading control. **b** The IR sensitization was only observed in Crispr-INPP4B cells, but not in shINPP4B cells. The three indicated cell lines were seeded into 3.5 cm plates at 3 × 10^5^ /plate. After one day of culture, cells were irradiated with 20 Gy of IR, survival cells were counted on day 3 post IR, the average survival rates were normalized to the untreated cells immediately prior to IR irradiation and plotted in **b**. *p < 0.05 indicates a significant difference of cell survival between Crispr-INPP4B and shINPP4B cells by one-way ANOVA test. **c** Knockdown of Akt in Crispr-INPP4B cells was confirmed by Western blot, shFF2 was used as a negative control. **d** Downregulation of Akt in Crispr-INPP4B A549 cells does not affect their IR sensitivity. The two indicated cell lines were cultured and treated as the same as described in **b** and cell survival rates were measured on day 3, *p* > 0.05 by Student’s *t* test.

It is known that INPP4B could suppress the activation of Akt^12–14^. Next, we tested whether there is a functional link between Akt activation and the DNA damage sensitization induced by loss of INPP4B. First, we transfected the CTL A549 cells with a MSCV-Hyg plasmid expressing a small hairpin RNA targeting INPP4B mRNA and generated an INPP4B knockdown cell line named as shINPP4B A549. CTL, shINPP4B and Crispr-INPP4B A549 cells were serum starved, insulin was added into those starved cells and the activation status of Akt was examined by Western blotting detecting phosphorylated S473 residue on Akt. Though the levels of phosphorylated Akt and the phosphorylated p70S6K, a downstream target of Akt-mTOR signaling, in shINPP4B cells, was similar to that detected in Crispr-INPP4B cells, but, the survival differences between shINPP4B and Crispr-INPP4B cells are significant post 20 Gy IR (*p* < 0.05) (Figs. 3A and 3B). These results suggest activation level of Akt unlikely contribute to the increased DNA damage sensitivity induced by INPP4B knockout. Moreover, Akt in the Crispr-INPP4B A549 cells was also knocked down by shRNA (Fig. 3C), and we compared the IR sensitivity between the resulting Akt knockdown cells with the Crispr-INPP4B A549 cells transfected with MSCV-FF2. Again, no obvious difference of IR sensitivity was observed between the two cell lines (Fig. 3D, *p* > 0.05), confirming the decreased Akt level in INPP4B knockout cells does not affect their IR sensitivity. Taken together, we conclude elevated Akt activation triggered by loss of INPP4B is not a contributor to the increased DNA damage sensitivity induced by loss of INPP4B in A549 cells.

### Homologous recombination repair is impaired upon loss of INPP4B

**Fig. 4:**
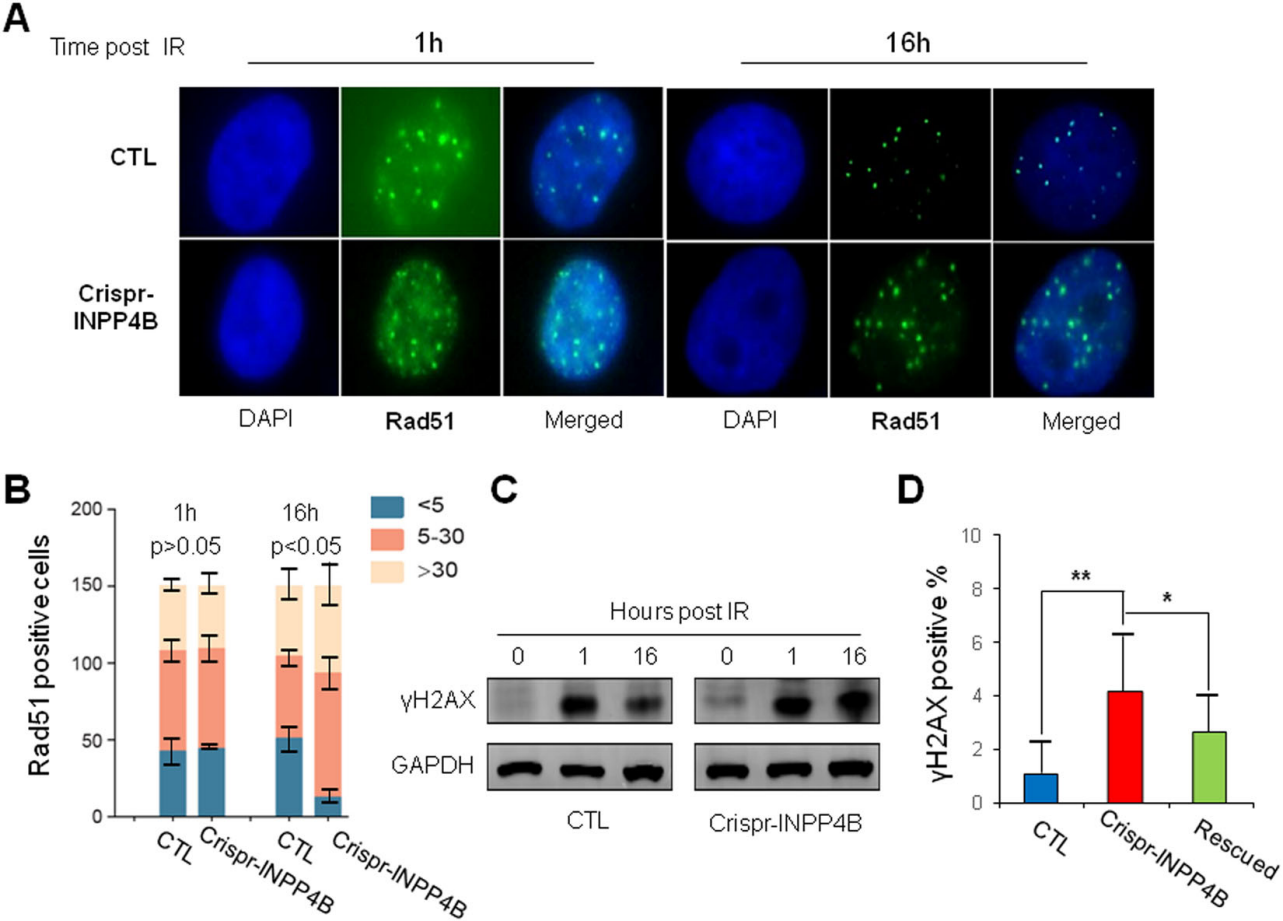
Prolonged γH2AX activation and Rad51 foci formation in Crispr-INPP4B A549 cells implicate impairment of HR repair. **a** The number and signal intensity of Rad51 foci remain high in Crispr-INPP4B A549 cells after 16 h of recovery post 10 Gy IR compared with CTL cells. At the indicated recovery time point, cells were fixed, permeabilized and processed for immunofluorescence staining using antibodies against Rad51. Nuclei were counterstained with DAPI. Representative foci formation images at the indicated time points were shown at a magnification of x1000. **b** Quantitative summaries of Rad51 foci. The foci numbers were counted in a total of 150 cells at 1 h and 16 h time points post IR by fluorescence microscopy. The cells were divided into 3 sub-groups by foci number: <5 per nucleus, 5–30 per nucleus, and >30 per nucleus. Error bars indicate the standard deviation occurred in each sub-group. Positive cells in 5–30 sub-group were statistically compared between CTL and Crispr-INPP4B cells by Student’s *t*-test. **c** Western blotting confirmed the signal of γH2AX was decreased in CTL A549 cells after 16 h of IR recovery while remained high in Crispr-INPP4B A549 cells. **d** A significant increase of spontaneous endogenous γH2AX foci was observed in the cells with loss of INPP4B expression. A total of 150 cells were observed and counted under fluorescence microscopy. The mean percentage of γH2AX foci positive cells in each cell line was plotted. Asterisk (*) indicates *p* < 0.05 and asterisks (**) indicates *p* < 0.01 by one-way ANOVA test.

The fact that PARP inhibitor olaparib can enhance the IR sensitivity in INPP4B knock out cells hints some levels of HR repair defects might exist in these cells. Next, we would like to determine the correlation between INPP4B expression and HR repair. In this regard, the γH2AX and Rad51 foci, two well-known markers for HR repair, were examined. We assessed the foci formation of γH2AX and Rad51 in CTL and Crispr-INPP4B cells treated with 10 Gy IR, and counted the foci at 1 h and 16 h time points post IR by fluorescence microscopy. At 1 h time point, the average number and signal intensity of γH2AX and Rad51 foci in CTL and Crispr-INPP4B cells were similar (Figs. 4A and S1). However, after 16 h of recovery, the average number and signal intensity of γH2AX and Rad51 foci in CTL cells were dropped significantly. In contrast, the foci number and signal intensity remained high in Crispr-INPP4B A549 cells (Figs. 4B and S1). Normally, after 16 h of recovery following 10 Gy IR, the repair of damaged DNA should almost have been accomplished. As a result, the DNA damage response signaling and foci formation of repair related proteins would diminish gradually. However, the persistent activation of γH2AX (Fig. 4C) and the prolonged Rad51 foci formation in Crispr-INPP4B cells after 16 h recovery of IR, strongly indicate the HR repair process is impaired upon loss of INPP4B. Moreover, a high level of spontaneous γH2AX foci occurred in Crispr-INPP4B A549 cells under normal cell culture condition also supports the positive correlation between INPP4B and HR repair (Fig. 4D).

Furthermore, we employed an artificial assay to confirm the HR repair impairment in the Crispr-INPP4B A549 cells. We integrated the genomes of CTL and Crispr-INPP4B A549 cells with a GFP-PEM1 HR reporter gene cassette by transfection, the resulting cells were co-transfected with two vectors expressing I-Sce I enzyme and orange fluorescence protein (OFP). Upon I-Sce I induction and cleavage of GFP-Pem1 gene, the cleaved GFP-Pem1 gene would convert into a full GFP gene through HR repair. As a result, GFP expression would be an accurate readout for determining HR repair capacity in I-Sce I induced cells^19^. The number of GFP positive cells was measured by FACS analysis and normalized to OFP positive cells for transfection control. At 48 h time point after induction of I-Sce I, the average percentages of GFP positive cells in CTL and Crispr-INPP4B cells are 14.82 ± 2.32% and 10.98 ± 1.90%, respectively (Fig. S2). The fewer GFP positive cells appeared in the Crispr-INPP4B cells suggest INPP4B expression is required for efficient HR repair in A549 cells. Lastly, the restored HR repair capacity in the recused cells expressing a Crispr-resistant INPP4B gene further confirmed the observed HR repair impairment is correlated with the loss of INPP4B (Fig. S2B).

### INPP4B responds to DNA damage by nuclear translocation

**Fig. 5:**
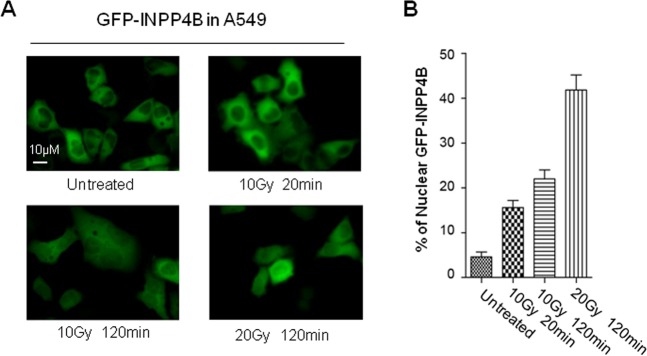
IR irradiation triggers INPP4B nuclear translocation in A549 cells. **a** A549 cells stably transfected with pEGFP-C1-INPP4B plasmid were irradiated with 10 or 20 Gy gamma-ray, the sub-cellular localization of GFP-INPP4B protein was observed by fluorescence microscopy at the indicated recovery time, the representative pictures were taken at magnification ×400. **b** Quantitative analysis of nuclear redistribution of GFP-INPP4B. Image J software was employed to quantify the nuclear signal intensity of GFP-INPP4B, as well as the whole cellular signal of GFP-INPP4B with normalization to the signal of GFP-INPP4B measured in the untreated cells. The proportion of nuclear GFP-INPP4B signal was calculated by the normalized nuclear GFP-INPP4B signal versus the normalized whole cellular GFP-INPP4B signal. At least 100 cells were measured under each condition. The average proportion of nuclear GFP-INPP4B signal in each condition was plotted in **b**. An error bar stands for standard deviation of nuclear INPP4B signal.

Above findings suggest INPP4B is somehow linked to DNA repair. How INPP4B responds to DNA damage is unclear so far. To answer this question, we generated an A549 cell line stably expressing GFP-INPP4B driven by pEGFP-C1 vector, subjected these GFP-INPP4B expressing cells to IR irradiation, and observed the subcellular localization of GFP-INPP4B by fluorescence microscopy. Under normal culture condition, GFP-INPP4B protein predominantly localizes in cytoplasm. Following IR irradiation, an increase of nuclear redistribution of GFP-INPP4B was observed in a time and IR dose dependent manner (Figs. 5A and 5B). We also examined the redistribution of endogenous INPP4B protein post IR irradiation. However, due to the high level of non-specific staining of the INPP4B antibody used for cell staining, we failed to draw a confirmative conclusion. Nevertheless, our results suggest ectopically expressed GFP-INPP4B protein is a DNA damage response protein by redistribution from cytoplasm to nucleus following IR irradiation.

### INPP4B interacts with Rad50 and is required for Rad50 stabilization in vivo

Next, we would ask how INPP4B is involved in the repair of DNA double-strand breaks. Since INPP4B may possess a dual phosphatase activity^22^, it could not only dephosphorylate phosphatidylinositol 3, 4-disphosphate, but also may interact with a number of protein substrates. In order to identify potential INPP4B interacting proteins, we performed immunoprecipitation by using an antibody against endogenous INPP4B protein in A549 cells, and subjected the immuno-precipitated protein complexes to mass spectrometry. By comparing with the results from an unrelated antibody, we identified a total of 107 proteins which are potential INPP4B interactors in A549 cells (Table S1).

**Fig. 6:**
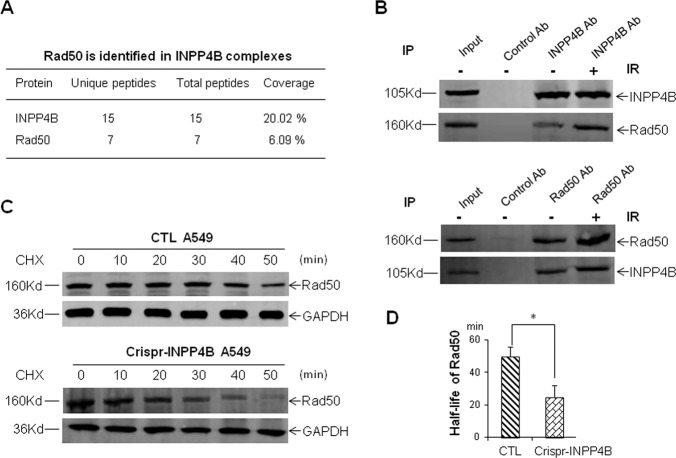
Interaction of INPP4B with Rad50 and destabilization of Rad50 upon loss of INPP4B. **a** Rad50 was identified from immuno-precipitated INPP4B complexes by mass spectrometry. **b** Reciprocal verification of the interaction between INPP4B and Rad50 in A549 cells. Two micrograms of antibodies against INPP4B or Rad50 were used for each reciprocal immunoprecipitation in a total of 2 mg of A549 cell lysates, and the precipitated proteins were resolved on 6% SDS–PAGE gel and detected with the indicated antibodies. In IR treatments, cells were harvested at 2 h post IR. **c** Representative Western blot images for measuring the stability of Rad50 in CTL and Crispr-INPP4B A549 cells. The half-life of Rad50 in each cell line was determined by densitometry scanning compared to the signal intensity at time zero with CHX treatment, and normalized to GAPDH signal. The average half-life of Rad50 was based on three independent CHX treatments and plotted in **d**, and data are presented as means ± SD (min). An error bar represents SD. Asterisk (*) indicates *p* < 0.05 by Student’s *t*-test.

Among these candidate proteins, Rad50, a component of Mre11-Rad50-Nbs1 complex crucial for sensing and repairing DNA double-strand breaks caused our attention (Fig. 6A)^23,24^. Reciprocal immunoprecipitation of each endogenous protein in A549 cells confirmed the interaction between INPP4B and Rad50 (Fig. 6B). Moreover, IR irradiation could enhance their mutual interaction (Fig. 6B), consistent with the nuclear translocation of INPP4B following IR. In order to elucidate the functional consequence underlying this interaction, we measured the Rad50 protein stability by using cycloheximide treatment. As shown in Figs. 6C and 6D, The half-life of Rad50 in CTL A549 cells is almost twice longer than that in Crispr-INPP4B A549 cells (*p* < 0.05), indicating loss of INPP4B leads to destabilization of Rad50 in vivo. We conclude INPP4B not only interacts with Rad50, but also is required for the maintenance of Rad50 stability in vivo. Thus, destabilization of Rad50 upon loss of INPP4B in A549 cells might result in the increased DNA damage sensitivity.

## Discussion

In this study, we found loss of INPP4B expression in A549 cells caused sensitization to IR irradiation, and INPP4B involved in HR repair by interacting with Rad50. Several lines of existing evidence support our findings. First of all, INPP4B is a DNA damage response protein, IR irradiation triggers cytoplasmic INPP4B redistribution into nucleus, consistent with the re-localization phenomena of many cytoplasmic proteins such as cyclic GMP-AMP synthase (cGAS)^25^, transcription regulators STATs^26^ and NF kappa B^27^. It has been shown cGAS rapidly translocates from cytoplasm to nucleus following DNA damage to interact with PARP1 and suppress HR repair^25^. STATs and NF kappa B are activated by phosphorylation in cytoplasm upon DNA damage and translocate into nucleus to initiate the transcription of their target genes. Secondly, loss of INPP4B protein sensitizes cells to IR irradiation and leads to prolonged activation of rH2AX and Rad51 foci formation. Moreover, INPP4B knockout cells also are sensitive to PARP inhibitor olaparib. Given the fact that endogenous DNA damage generated by PARP inhibition is well-tolerated in normal cells because of functional compensation from HR mediated repair^20^, our findings pointed to an intrinsic DNA repair defect occurred in A549 cells with loss of INPP4B protein. Thirdly, the DNA damage sensitivity of breast cancer cell line MDA-MB-231, leukemia cell line KG-1 and ovarian cancer cell line Ovca429 has been demonstrated to be affected by the expression level of INPP4B^16–18^. In addition, INPP4B was shown to affect the stability of ATM-BRCA1 complex in human 293T cells^18^. In line with this previous finding, we found INPP4B interacts with Rad50 in vivo and loss of INPP4B results in decreased stability of Rad50. Similarly, PTEN has been reported to interact with a number of nuclear localized proteins to maintain genome integrity^4,28^, those functions are dependent on the nuclear re-localization of PTEN rather than the lipid phosphatase activity associated with PTEN^29^. Whether this is the case for INPP4B requires more vigorous investigations in the future.

**Fig. 7:**
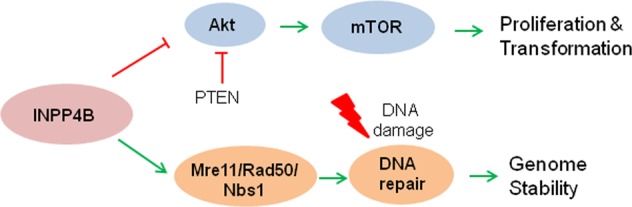
A dual role model for the cellular functions of INPP4B. INPP4B exerts its antitumor activity through suppression of Akt-mTOR signaling, as well as promotion of Rad50 mediated DNA double strand break repair. A green line with an arrow indicates activation and a red line with a slash depicts inhibition. For more details see the related text in the discussion section.

Despite the fact that INPP4B is known for suppression of tumorigenesis by inhibiting PI3K-Akt-mTOR signaling, our study revealed a new role of INPP4B in regulating DNA repair process. Thus, we propose a dual role model for the cellular functions of INPP4B as depicted in Fig. 7. Under normal condition, INPP4B works in coordination with PTEN to suppress PI3K-Akt-mTOR signaling. Upon genotoxic stress, INPP4B may interact with Rad50 and lead to stabilization of Rad50 to facilitate sensing and repair of DNA double-strand breaks.

Rad50, originally identified in yeast genetic screen with increased the sensitivity to ultraviolet light^23^, is a key component in homologous recombination pathways. Through interaction with Mre11 and Nbs1, Rad50 forms the MRN complex to tether linear DNA double-strand breaks^30^. Moreover, Rad50 involves in the earliest events in DNA damage response by sensing double-strand breaks and activating ATM, ATR and downstream signaling cascades^23,24^. Reduced stability of Rad50 could lead to impaired DNA double-strand break resection in both *Schizosaccharomyces pombe and Saccharomyces cerevisiae*^24^. How INPP4B impacts tethering and resection of DNA double-strand breaks via promotion of Rad50 stability remains unclear.

Numerous previous findings indicate RAD50 upregulation is associated with poor clinical outcomes of radiotherapy for treatment of lung, colorectal and breast cancers^31–33^. Targeting DNA repair proteins such as Rad50 in cancer cells would offer great advantages over conventional radiotherapy or chemotherapy^34^. Our finding that destabilization of Rad50 induced by loss of INPP4B may reveal the Achilles’ heel of certain cancers. In fact, our finding that INPP4B knockout A549 cells are sensitive to DNA repair inhibitor olaparib further supports the scenario of defected DNA repair could be exploited for cancer therapy. Therefore, our findings would offer targeting INPP4B deficiency or defects as a promising therapeutic strategy for personalized cancer treatment in the clinic.

In conclusion, our study has revealed an unanticipated role of tumor suppressor INPP4B in maintenance of genome integrity by facilitating Rad50 mediated DNA double-strand break repair, provided a new therapeutic strategy for INPP4B-based therapy for cancer treatment.

## Supplementary information

Supplemental Figure Legends

Supplemental Figure S1

Supplemental Figure S2

Supplemental Table S1
