# Supplementary material for: Loss of tumor suppressor inositol polyphosphate 4-phosphatase type B impairs DNA double-strand break repair by destabilization of DNA tethering protein Rad50
Source: Cell Death Dis. 2020 Apr 27;11(4):292. doi: 10.1038/s41419-020-2491-3 (PMC7184567; doi:10.1038/s41419-020-2491-3)
Supplement: Supplementary file 1 — Supplemental Figure Legends [file 41419_2020_2491_MOESM1_ESM.docx]

**Supplementary Figure Legends**

**Supplementary Figure S1**

**Prolonged γH2AX** **foci formation and retention in cells with loss of INPP4B following IR irradiation.**

The number and signal intensity of γH2AX foci are still high in Crispr-INPP4B A549 cells after 16h of recovery post 10Gy IR compared with CTL cells. Nuclei were counterstained with DAPI. The representative foci formation images at indicated time points were shown at a magnification of x1000.

**Supplementary Figure S2**

**A GFP reporter assay further confirms loss of INPP4B leads to impairment of DNA homologous recombination repair.**

**(A)** An artificial GFP reporter assay for HR repair was performed. CTL, Crispr-INPP4B and rescued A549 cells stably integrated with GFP –Pem1 plasmids were transiently co-transfected with vectors expressing I-Sce I and orange fluorescence protein (OFP used for normalization of transfection efficiency). After 48h of I-Sce I induction, GFP cells were observed under fluorescence microscopy and representative images were shown in (A). **(B)** Cells were harvested by trypsin digestion and subjected to cell sorting by flow cytometry. GFP positive cells from three independent cell sorting experiments were normalized to OFP expression and summarized in (B). Data are presented as means ±SD. An error bar depicts SD. * indicates p< 0.05 and ** indicates p< 0.01 by one-way ANOVA test between indicated cells.
