# Supplementary figures and images for: Loss of tumor suppressor inositol polyphosphate 4-phosphatase type B impairs DNA double-strand break repair by destabilization of DNA tethering protein Rad50
Source: Cell Death Dis. 2020 Apr 27;11(4):292. doi: 10.1038/s41419-020-2491-3 (PMC7184567; doi:10.1038/s41419-020-2491-3)

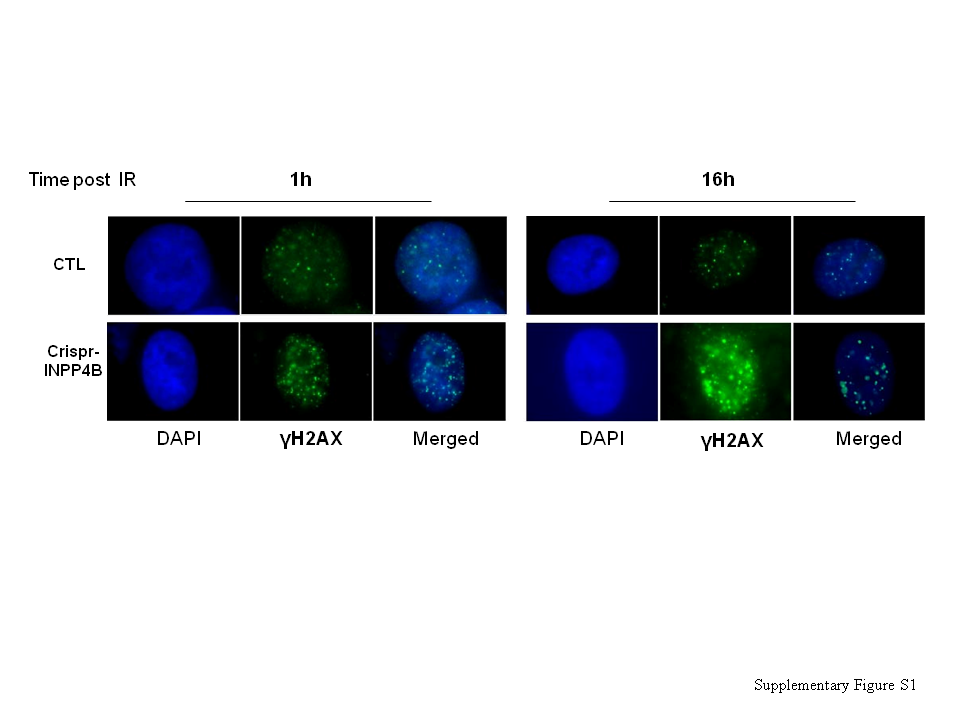

Supplement: Supplementary file 2 — Supplemental Figure S1 [file 41419_2020_2491_MOESM2_ESM.tif]

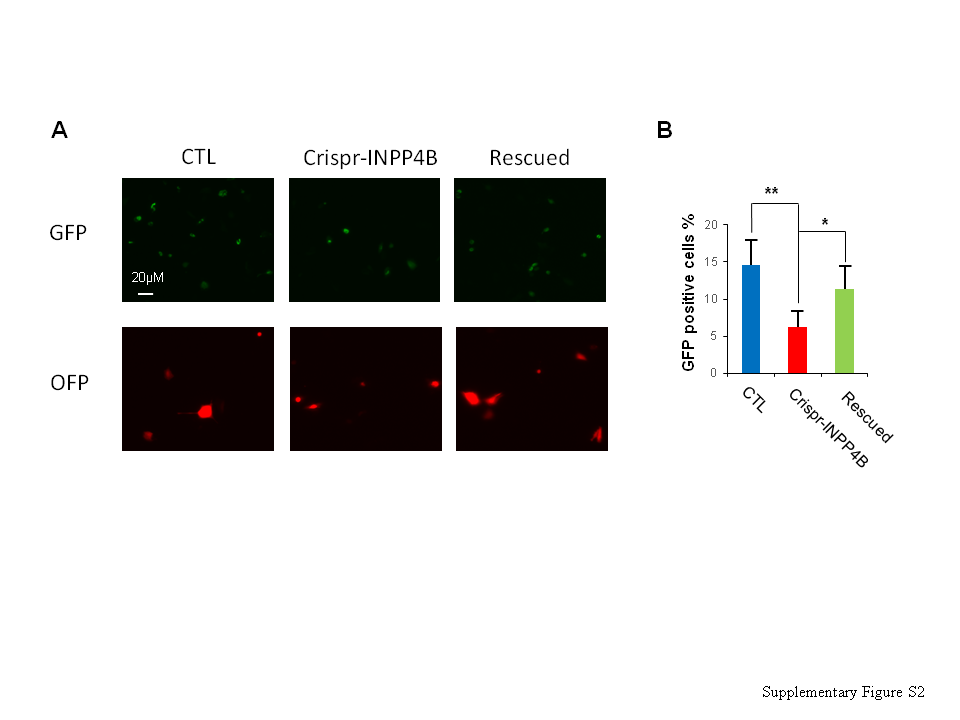

Supplement: Supplementary file 3 — Supplemental Figure S2 [file 41419_2020_2491_MOESM3_ESM.tif]
